# Isolation, identification, and slow-growth phenotype characterization of a clinically derived *Escherichia coli* from urinary tract infection

**DOI:** 10.1371/journal.pone.0358948

**Published:** 2026-10-29

**Authors:** Wenqiang Yu, Yao Ou, Shanshan Dong, Xiangyu Deng, Siqiang Niu, Xiongyu Jiang, Ranran Tang, Shuqi Zhang, Qiongyuan Zhang, Dinghua Liu, Chunhong Zou, Jiangling Wu, Yumei Zhao

**Affiliations:** 1 Department of Clinical Laboratory, University-Town Hospital of Chongqing Medical University, Chongqing, China; 2 College of Laboratory Medicine, Chongqing Medical University, Chongqing, China; 3 Department of Laboratory Medicine, The First Affiliated Hospital of Chongqing Medical University, Chongqing, China; 4 Second Clinical College, Chongqing Medical University, Chongqing, China; Tribhuvan University, NEPAL

## Abstract

A slow-growth *Escherichia coli* isolate, designated *E. coli* 105X, was recovered from a patient with urinary-tract infection. Initial biochemical profiling misidentified it as *Shigella boydii*; however, matrix-assisted laser desorption/ionization time-of-flight mass spectrometry and average nucleotide identity analysis (98.67% relative to uropathogenic *E. coli* CFT073) unequivocally reclassified the strain as *E. coli*. Antimicrobial susceptibility testing revealed broad-spectrum resistance to β-lactams, aminoglycosides and fluoroquinolones, which was mediated by 53 acquired resistance determinants, including the extended-spectrum β-lactamase genes *bla*_CTX-M-55_ and *bla*_TEM-1_, together with an expanded repertoire of efflux pump systems. Whole-genome sequencing (WGS) disclosed 45399 single-nucleotide polymorphisms and 1736 insertions/deletions relative to *E. coli* CFT073. Functional enrichment analyses demonstrated that non-synonymous mutations in 11 flagellar biosynthesis genes (e.g., *flgA*, *fliK*), the peptidoglycan synthase-encoding *mrcB*, and 29 growth-associated loci collectively perturb energy allocation and compromise biofilm formation, resulting in a markedly prolonged doubling time. Concurrently, *E. coli* 105X lacks canonical virulence factors such as the siderophore enterobactin and P fimbriae, but has acquired temperature-sensitive hemagglutinin and type IV pili. Taken together, *E. coli* 105X exemplifies a distinctive adaptive strategy in which metabolic bottlenecks synergize with structural mutations to establish a “slow-growth, high-resistance, re-engineered virulence” phenotype. These findings underscore the critical necessity of molecular diagnostic tools for accurate identification of morphologically atypical clinical *E. coli* isolates, and provide a theoretical rationale for developing targeted combination therapies that interfere with core bacterial energy-metabolism pathways.

## Introduction

*Escherichia coli* is a Gram-negative, non-spore-forming rod equipped with peritrichous flagella that confer motility, and it constitutes the dominant facultative anaerobe of the human intestinal microbiota. It is a leading cause of diverse infections in humans and animals, including urinary-tract infections, enteric diseases, and systemic infections [1,2]. Its marked phenotypic heterogeneity and rapid evolution of antimicrobial resistance pose substantial challenges for accurate diagnosis and infection control.

Conventional identification relies on biochemical profiling; however, *E. coli* and the genus *Shigella* share >99% 16S rRNA sequence similarity, resulting in a 15–30% misidentification rate [3,4]. Recent advances in molecular technologies, namely matrix-assisted laser desorption/ionization time-of-flight mass spectrometry (MALDI-TOF MS) and whole-genome sequencing (WGS), have demonstrated that *E. coli* and *Shigella* spp. represent distinct ecotypes of the same biological species, with average nucleotide identity (ANI) values exceeding 96% [5–7]. Therefore, routine laboratories report frequent biochemical misclassification, underscoring the limitations of traditional methods and suggesting that slow-growth strains may further confound phenotypic identification through metabolic pathway rewiring [8].

Slow-growth phenotypes, operationally defined as a significant prolongation of doubling time and reduction in overall biomass yield relative to wild-type or reference strains under identical culture conditions [8,9], have been linked to dysregulation of metabolic control networks. Mutations within the ATP-binding motif of the DEAD-box helicase DbpA impair ATPase activity. For instance, the arginine-to-alanine substitution R331A causes dominant-negative interference with the wild-type protein function. Consequently, cells exhibit pronounced colony morphotype alterations [10,11]. Moreover, aberrant assembly of the cell-cycle control complex disrupts the coordination between DNA replication and cytokinesis, driving the bacterium into a trade-off state between metabolic maintenance and growth [12,13]. Collectively, these mechanisms confer unique adaptive advantages upon slow-growth strains, yet the co-evolutionary dynamics linking slow growth with antimicrobial resistance and virulence remain poorly defined.

In this study, we isolated a slow-growth *E. coli*, designated *E. coli* 105X, from a patient with urinary-tract infection. Compared with typical *E. coli*, *E. coli* 105X exhibited markedly reduced growth and biochemical profiles indistinguishable from *Shigella boydii*. Using comprehensive WGS, we characterized the genetic determinants underlying the slow-growth phenotype and delineated their interplay with antimicrobial resistance and virulence evolution. Our findings provide a molecular framework for accurate clinical identification and for precise control strategies against multidrug-resistant *E. coli*.

## Materials and Methods

### Clinical strain acquisition and primary isolation

In October 2023, a slow-growth *E. coli* strain (*E. coli* 105X) was isolated from the urine of a 27-year-old male patient with urinary tract infection at University Town Hospital, Chongqing Medical University. The patient presented with chills, rigors, and fever; abdominal CT indicated nephroureterolithiasis with hydronephrosis and concurrent infection, alongside markedly elevated CRP and PCT. Urine specimens were Gram-stained, microscopically examined, and aerobically cultured on blood and MacConkey agar for 24 h at 37°C. Pure single colonies (white, circular, smooth, moist) were obtained through three-sector streaking and sub-cultured for ten passages on blood agar to ensure clonal purity. This study was approved by the Ethical Review Committee of University-Town Hospital of Chongqing Medical University (protocol No. LL-202323). Written informed consent was obtained from the participant after full explanation of the study procedures.

### Phenotypic and molecular identification

Colonies were identified via the BD Phoenix™ automated system after suspension in 0.45% saline (0.50–0.63 McFarland). Serological typing was performed by slide agglutination using 0.45% saline suspensions of the isolate and commercial *Shigella* antisera (Tianrun, Ningbo, China; Cat. No. TR201). The antisera panel included polyvalent antisera covering *Shigella* dysenteriae (groups 1–4, 5–8, 9–12), *Shigella* flexneri (serotypes I–VI and X/Y variants), *S. boydii* (groups 1–5, 6–10, 11–14, 15–18), and *Shigella sonnei* (phases 1 and 2), as well as corresponding monovalent antisera targeting individual O-antigens of each serotype. Agglutination was assessed macroscopically within 1 min, with physiological saline serving as a negative control. MALDI-TOF MS was performed by QuanID Microbial Identification System (Rongzh, Qingdao, China). For sample preparation, pure colonies of the target strains were picked and uniformly smeared onto the target plate. Subsequently, 2 µL of B reagent (Rongzhi, Qingdao, China, Cat. No. QT-SJ01-L) was dispensed onto the smear and allowed to air dry. Finally, 1.5 µL of A reagent was added to the same spot, air dried, and the plate was subjected to spectral acquisition. The acquired mass spectra were analyzed and identified using the dedicated software provided with the instrument. The identification results were generated by comparing the obtained spectra against an in-house curated database specific to clinical pathogens.

### Growth kinetics and antimicrobial susceptibility

Growth curves were constructed in beef-peptone-glucose medium (37 °C, 180 rpm) by measuring OD_600_ every 2 hours, with sterile medium incubated in parallel as a blank control. Susceptibility testing was carried out with the BD Phoenix™ automated system. MICs were determined by broth microdilution per CLSI guidelines and interpreted using CLSI M100-2025 breakpoints [14], with *E. coli* ATCC 25922 as the quality control strain.

### Genomic DNA extraction and sequencing

Overnight Luria-Bertani (LB) broth cultures (37 °C, 180 rpm) were processed using the Hieff NGS® MaxUp II DNA Library Prep Kit for Illumina®. DNA quality (Qubit™ dsDNA HS Assay) and integrity (Agilent 2100 Bioanalyzer) were assessed before paired-end sequencing on Illumina NovaSeq. Raw reads were quality-trimmed and filtered using Fastp (version 0.11.2). Briefly, adapter sequences were automatically detected and trimmed; global trimming was applied to the 5′ and 3′ ends of each read; sliding-window trimming was performed with a window size of 4 bp, discarding any window with mean base quality below Q20; base correction was conducted based on read-overlap consensus; and low-quality reads—defined as those containing >40% bases with Phred quality scores < Q15 or with a post-trimming length < 35 nt—were removed. Hybrid assembly of short- and long-read data employed SPAdes v3.5.0 and Canu v1.3 [15,16].

### Evolutionary relationship and phylogenomic analysis

Whole-genome CDSs were aligned with MAFFT; phylogenetic trees were inferred with FastTree. Pairwise ANI values were calculated via the JSpeciesWS web server for taxonomic confirmation. Functional annotation used Prokka v1.10 and the NCBI PGAP pipeline to catalog resistance, virulence, and metabolic genes [17]. Core- and pan-genome analyses were performed with Roary v3.11.2 against closely related reference strains [18].

## Results

### Isolation and Identification of *E. coli* 105X

On blood agar, *E. coli* 105X produced highly translucent colonies with a mean diameter of ~1 mm, markedly smaller than the 6-mm colonies of the control strain *E. coli* ATCC 25922 (Fig 1A,1B). Biochemical profiling showed *E. coli* 105X to be positive for L-leucine-7-amido-4-methyl-coumarin and negative for L-arabinose, L-arginine-7-amido-4-methyl-coumarin, galacturonic acid, acetate, malonate, ornithine, p-nitrophenyl phosphate, L-rhamnose, N-acetyl-glucosamine, and citrate. This biochemical pattern showed a closer match to the standard biochemical fingerprint of *S. boydii* (S1 Table). Serological testing with *Shigella* antisera yielded no agglutination, indicating the absence of *Shigella*-specific antigens (S1 Fig). MALDI-TOF MS unequivocally identified the isolate as *E. coli* (S2 Fig). The quantitative analysis of growth curves using the logistic growth model (GrowthCurver package) revealed that *E. coli* 105X had a significantly lower growth rate (r = 0.226 h^−1^) compared to *E. coli* ATCC 25922 (r = 0.294 h^−1^), representing a 23.1% reduction. Additionally, the area under the curve (AUC) for *E. coli* 105X (3.439) was 31.8% lower than that of *E. coli* ATCC 25922 (5.040), indicating reduced overall growth capacity (Fig 1C).

**Fig 1:**
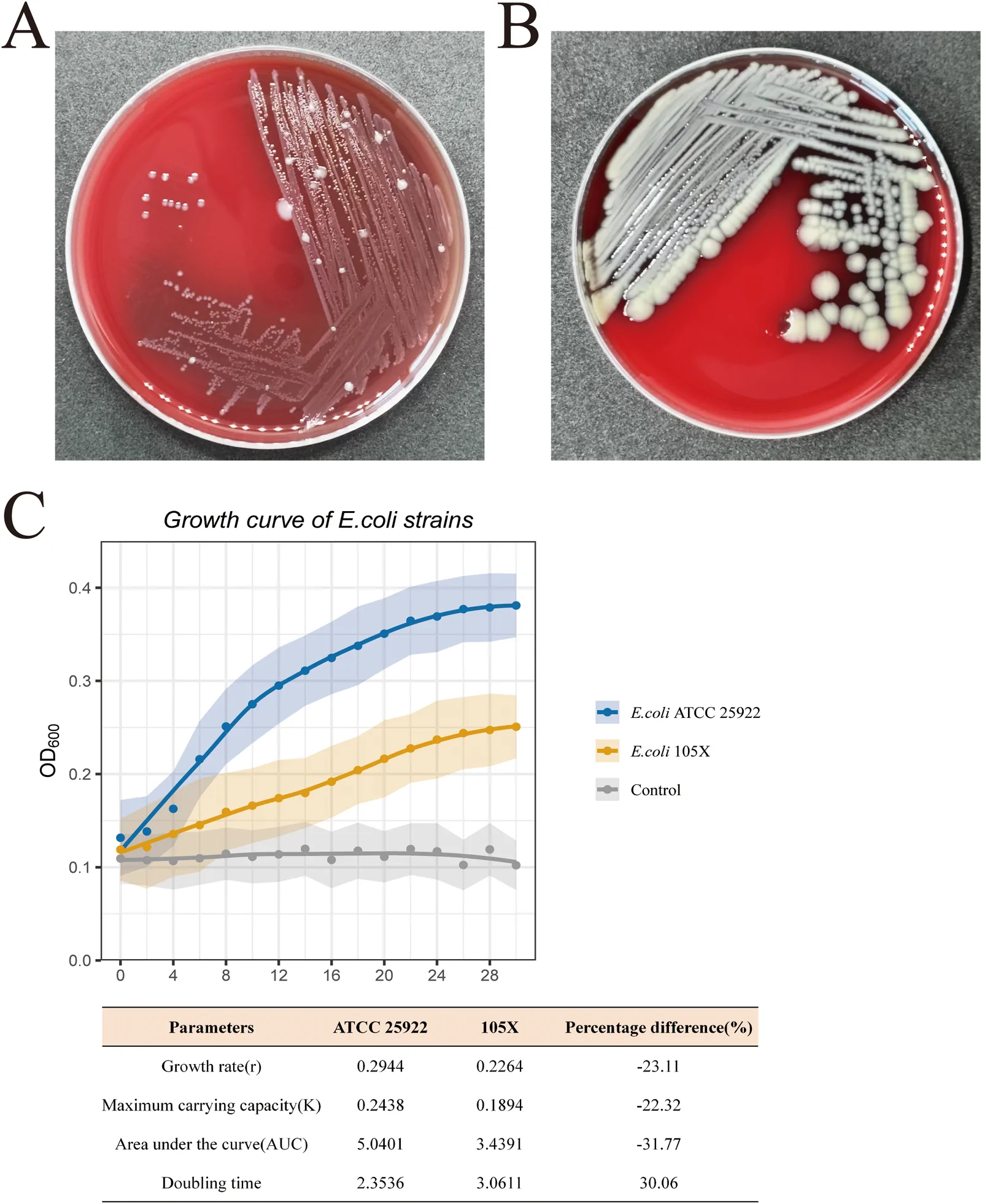
Growth characteristics of *E. coli* 105X. **(A)** Colony morphology of *E. coli* 105X cultured on blood agar medium. **(B)** Colony morphology of parallel subcultured quality control strain *E. coli* ATCC 25922 on blood agar. **(C)** Comparison of the growth curves between *E. coli* 105X and *E. coli* ATCC 25922.

### Antimicrobial susceptibility testing

The broth microdilution susceptibility testing showed that *E. coli* 105X was resistant to Amikacin (MIC > 16 µg/mL), gentamicin (MIC > 8 µg/mL), cefazolin (MIC > 16 µg/mL), cefotaxime (MIC > 32 µg/mL), cefepime (MIC = 16 µg/mL), aztreonam (MIC > 16 µg/mL), ampicillin (MIC > 16 µg/mL), piperacillin (MIC > 64 µg/mL), ciprofloxacin (MIC > 2 µg/mL), Levofloxacin (MIC > 8 µg/mL), and Moxifloxacin (MIC > 4 µg/mL). Ceftazidime was intermediate (MIC = 8 µg/mL) (Table 1).

**Table 1:** Antimicrobial susceptibility testing results for *E. coli* 105X.

Antibiotics	^a^MIC (μg/mL)	^b^Final SIR
Amikacin	>16	R
Gentamicin	>8	R
Imipenen	<=1	S
Meropenem	<=1	S
Cefazolin	>16	R
Ceftazidime	8	I
Cefotaxime	>32	R
Cefepime	16	R
Aztreonam	>16	R
Ampicillin	>16	R
Piperacillin	>64	R
Amoxicillin-Clavulanate	8/4	S
Ampicillin-Sulbactam	8/4	S
Piperacillin-Tazobactam	<=4/4	S
Colistin	<=0.5	S
Trimethoprim-Sulfameth oxazole	<=0.5/9.5	S
Chloramphenicol	8	S
Ciprofloxacin	>2	R
Levofloxacin	>8	R
Moxifloxacin	>4	R
Tetracycline	<=2	S

^a^ MIC: Minimum Inhibitory Concentration.

^b^ Final SIR: S: Susceptible R: Resistant I: Intermediate.

### General genomic characteristics and phylogeny

After trimming raw reads with Fastp, Illumina-based sequencing revealed that the slow-growth *E. coli* 105X possesses a genome of 4,894,031 bp with a GC content of 50.9% and 97.86% of bases achieving a Q20 quality score or higher. Annotation via the NCBI Prokaryotic Genome Annotation Pipeline (PGAP) predicted 4,890 protein-coding genes, spanning 4,476,981 bp (87.948% of the genome) with a mean length of 916 bp (S2 Table).

Functional annotation against the COG database classified 4,034 proteins into functional categories. The most abundant classes were carbohydrate transport and metabolism (G) and amino-acid transport and metabolism (E), together accounting for 17.70% of assigned proteins. Additional highly represented functional modules included energy production and conversion (C, 7.29%), transcription (K, 7.19%), cell wall/membrane/envelope biogenesis (M, 6.00%), inorganic ion transport and metabolism (P, 5.55%), translation/ribosomal structure and biogenesis (J, 4.61%), and replication/recombination/repair (L, 4.54%) (Fig 2A).

**Fig 2:**
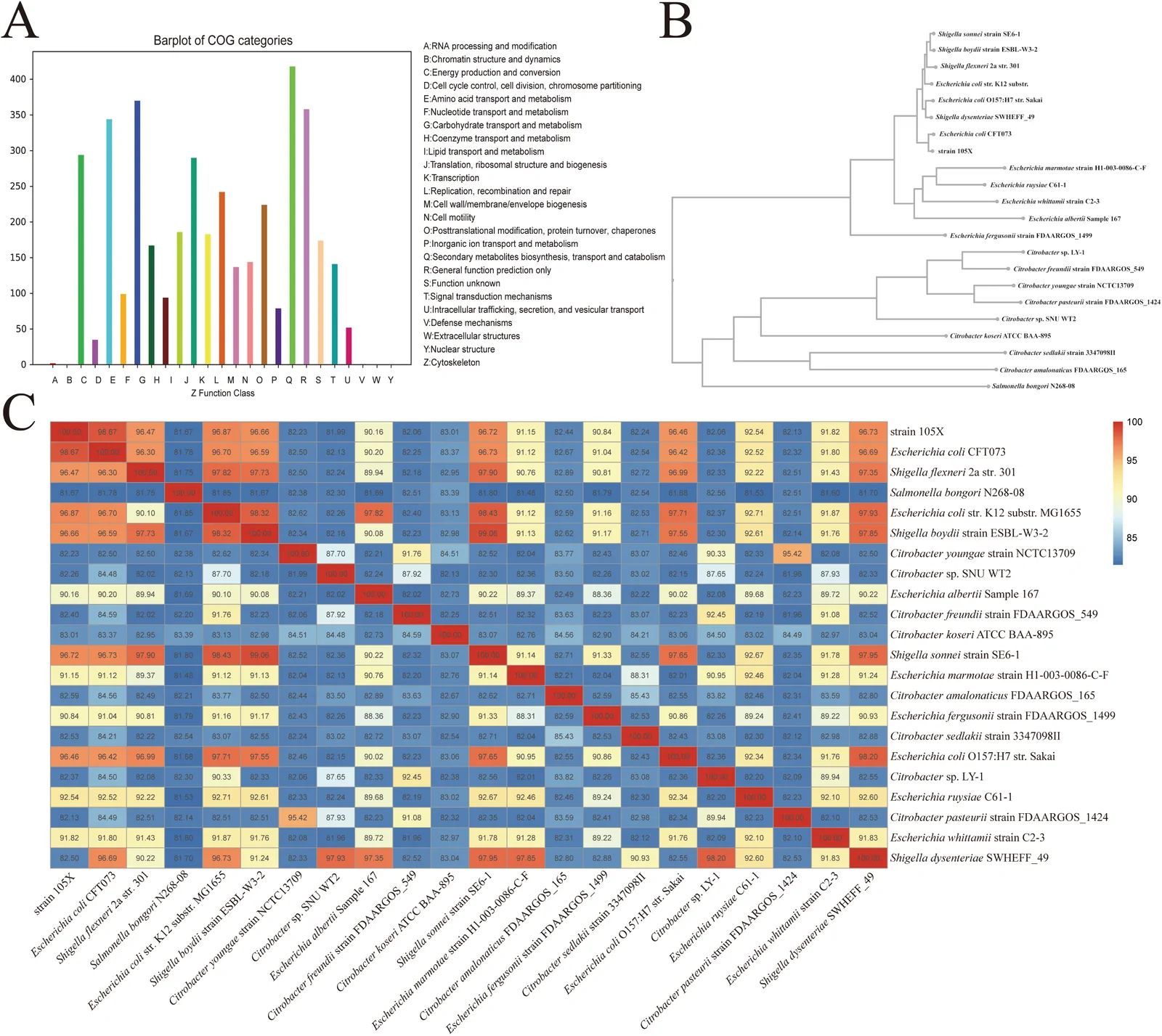
General genomic and phylogenetic characteristics of *E. coli* 105X. **(A)** COG functional classification bar chart of *E. coli* 105X genes. **(B)** Phylogenetic tree based on single-nucleotide polymorphisms (SNPs). **(C)** Heatmap displaying the ANI between the phylogenetically close species. The color bar on the right indicates the ANI value calculated using fastANI.

To delineate the precise genetic divergence of *E. coli* 105X, we constructed a whole-genome SNP-based phylogeny and calculated ANI. The phylogenetic tree placed *E. coli* 105X in the same clade as *E. coli* CFT073, *E. coli* O157:H7 Sakai, *S. dysenteriae* SWHEFF 49, *S. flexneri* 2a str. 301, *E. coli* K-12 MG1655, *S. boydii* ESBL-W3-2, and *S. sonnei* SE6−1 (Fig 2B). ANI values determined by fastANI corroborated this topology, with the highest identities observed for *E. coli* CFT073 (98.67%), *E. coli* K-12 MG1655 (96.86%), *S. dysenteriae* SWHEFF 49 (96.72%), *S. sonnei* SE6−1 (96.71%), *S. boydii* ESBL-W3-2 (96.66%), *E. coli* O157:H7 Sakai (96.54%), and *S. flexneri* 2a str. 301 (96.44%), thereby confirming the phylogenetic placement (Fig 2C).

### Enrichment Analysis and Prediction of Resistance and Virulence Determinants

Using *E. coli* CFT073 as the reference, we identified 45,399 SNPs and 1,736 indels in *E. coli* 105X. After excluding synonymous changes, 2,284 genes harbored non-synonymous mutations. GO enrichment analyses showed that these mutated genes were significantly over-represented in biological processes related to the metabolism of organic acids, alcohols, and amino acids (Fig 3A). At the cellular-component level, they were enriched in the cytoplasmic membrane, periplasmic space, and transporter complexes, implying roles in membrane architecture and substrate transport. For molecular functions, enrichment was evident in ATP binding, oxidoreductase activity, and transferase activity, reflecting alterations in cellular energy metabolism, redox homeostasis, and phosphoryl-group transfer. KEGG pathway enrichment further demonstrated that the mutated genes are enriched in four principal areas (Fig 3B). First, antimicrobial resistance: cationic antimicrobial peptide (CAMP) resistance and β-lactam resistance pathways. Second, metabolic reprogramming: lysine biosynthesis, glycine–serine–threonine metabolism, and glutamate/aspartate/alanine metabolism. Third, DNA repair: base-excision repair, suggesting compromised genomic stability. Fourth, motility and chemotaxis: bacterial chemotaxis and flagellar assembly pathways, indicating potential defects in motility and surface adherence.

**Fig 3:**
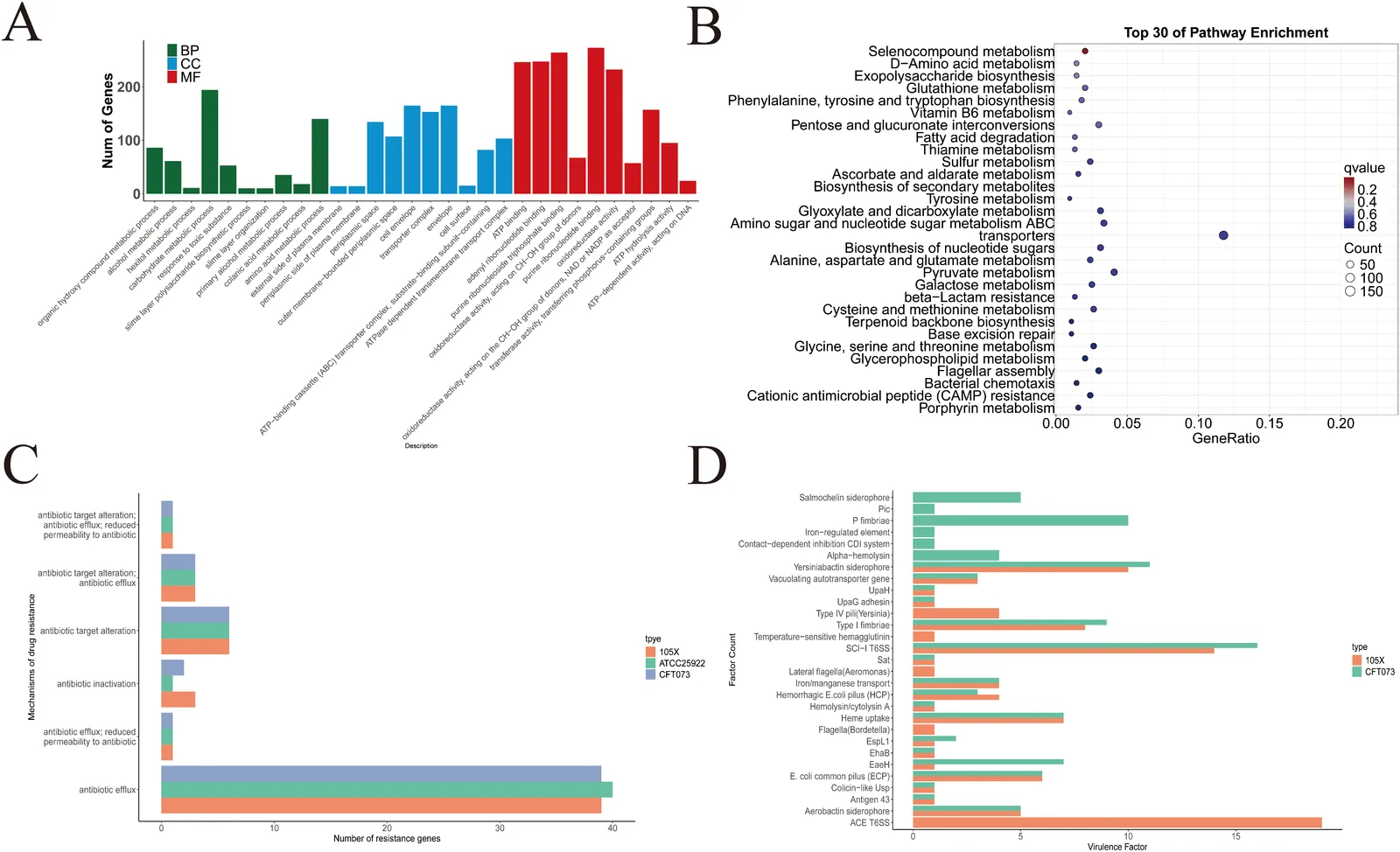
Enrichment analysis and prediction of resistance and virulence determinants. **(A)** The mutated genes of *E. coli* 105X were analyzed for enrichment in three GO ontologies: biological process (BP), cellular component (CC), and molecular function (MF). The top 10 enriched GO terms sorted by number were visualized as bar graph. **(B)** Top 30 KEGG pathways sorted by predicted Q-value for mutated genes of *E. coli* 105X in each strain. **(C)** Comparison of antimicrobial resistance genes in *E. coli* 105X, *E. coli* ATCC 25922 and *E. coli* CFT073. **(D)** Comparison of virulence factors in *E. coli* 105X and *E. coli* CFT073.

The entire genome of *E. coli* 105X was uploaded to the Comprehensive Antibiotic Resistance Database (CARD) for resistance gene identification. The results showed that the *E. coli* 105X genome carries 58 resistance genes, which confer resistance via four mechanisms: antibiotic efflux, antibiotic inactivation, target modification, and reduced permeability (Table 2). It comprises 43 genes (81%) encoding antibiotic efflux pumps. These include 21 resistance–nodulation–division (RND) family components such as *acrA*, 13 major facilitator superfamily (MFS) transporters exemplified by *mdtH*, 2 ATP-binding cassette (ABC) systems, and the small multidrug resistance (SMR) pump *emrE*. In addition, four transcriptional regulators (*marA*, *soxS*, *leuO*, and *kdpE*) reduce permeability. Twelve genes modify antibiotic targets, including *pmrF*, *bacA*, *eptA*, *ugd*, *arnT*, *gyrA*, *tufA*, and *vanG*. Five genes mediate antibiotic inactivation, including *aph(4)-Ia*, *bla*_TEM⁃1_, *aac(3)-IV*, *bla*_CTX-M-55_ and *bla*_EC-5_. Compared with *E. coli* CFT073, *E. coli* 105X carries an additional inactivation determinant (*bla*_CTX-M-55_); relative to *E. coli* ATCC 25922, it has acquired both ESBL genes (*bla*_CTX-M-55_ and *bla*_TEM⁃1_) and lost the *mdtM* efflux locus (Fig 3C).

**Table 2:** Classification of antimicrobial resistance genes identified in *E. coli* 105X based on functional resistance mechanisms.

Mechanisms of antimicrobial resistance	Antimicrobial resistance gene(s)
ATP-binding cassette (ABC) antibiotic efflux pump	*yojI*, *msbA*
Major facilitator superfamily (MFS) antibiotic efflux pump	*mdtH*, *mdtG*, *mdtN*, *emrB*, *kpnF*, *kpnE*, *mdfA*, *mdtP*, *mdtO*, *emrA*, *emrR*, *emrK*, *emrY*
Resistance-nodulation-cell division (RND) antibiotic efflux pump	*acrA*, *acrB*, *acrD*, *acrE*, *acrF*, *acrS*, *acrR*, *baeR*, *cpxA*, *crp*, *gadX*, *gadW*, *marR*, *mdtA*, *mdtB*, *mdtC*, *mdtE*, *mdtF*, *oqxA*, *marA*, *rsmA*
Small multidrug resistance (SMR) antibiotic efflux pump	*emrE*
Major facilitator superfamily (MFS) antibiotic efflux pump, resistance-nodulation-cell division (RND) antibiotic efflux pump	*hns*, *evgA*, *evgS*
ATP-binding cassette (ABC) antibiotic efflux pump, major facilitator superfamily (MFS) antibiotic efflux pump, resistance-nodulation-cell division (RND) antibiotic efflux pump	*soxS*, *soxR*, *tolC*
Reduced antibiotic permeability	*marA*, *soxS*, *leuO*, *kdpE*
Alteration of antibiotic target	*pmrF*, *bacA*, *eptA*, *ugd*, *marR*, *acrR*, *soxS*, *soxR*, *gyrA*, *tufA*, *arnT*, *vanG*
Antibiotic inactivation	*aph(4)-Ia*, *bla*_TEM⁃1_, *aac(3)-IV*, *bla*_CTX-M-55_, *bla*_EC-5_

Whole-genome comparison of *E. coli* 105X and *E. coli* CFT073 within the Virulence Factors of Pathogenic Bacteria (VFDB) revealed marked differences in their virulence repertoires. *E. coli* 105X completely lacks enterobactin (salmochelin), P fimbriae and α-haemolysin, and carries fewer copies of yersiniabactin, type I fimbriae, the SCI-I type VI secretion system, EspL1 and EaeH. Conversely, the haemorrhagic *E. coli* fimbrial locus is expanded, while *E. coli* 105X uniquely harbours temperature-sensitive haemagglutinin, *Yersinia*-type IV pili, lateral flagella (*Aeromonas hydrophila* origin), *Bordetella*-type flagella and the ACE type VI secretion system (Fig 3D).

### Slow-growth-associated genes

Comprehensive GO annotation revealed 29 genes whose nonsynonymous variants are linked to the slow-growth phenotype in *E. coli* 105X. These include one metabolic gene (*frdB*). Eleven flagellar genes (*flgA*, *flgD*, *flgK*, *flgJ*, *flgN*, *fliK*, *fliT*, *fliH*, *fliS*, *fliR*, *flhA*) encode structural and regulatory components of the flagellar basal body and motor. Twelve fimbrial genes (*yadL*, *htrE*, *csgF*, *csgB*, *ydeR*, *ydeS*, *fimA*, *fi*mD, *fimF*, *fimH*, *fimI*, *yehB*) are involved in type I fimbriae and curli biosynthesis. Additionally, there is one peptidoglycan-synthetic gene (*mrcB*), three toxin–antitoxin system genes (*hipA*, *yhaV*, *prlF*), and one further gene (*hofN*) (S3 Table).

## Discussion

In the present study, a clinically derived *E. coli* strain designated *E. coli* 105X was isolated from a urinary tract infection. *E. coli* CFT073 is a prototype uropathogenic *E. coli* (UPEC) originally isolated from the blood and urine of a woman suffering from acute pyelonephritis; it elaborates a diverse array of toxins and enterotoxins and is a well-established model for studies of urinary-tract infections, bacteraemia and pyelonephritis [19,20]. This strain, *E. coli* 105X, exhibited the highest average nucleotide identity (ANI) when compared to the internationally recognized quality control reference strain CFT073 (Fig 2C). Therefore, *E. coli* CFT073 was selected as the control strain for subsequent comparative analysis. *E. coli* ATCC strain 25922 is widely utilized as a standard quality-control organism in clinical microbiology laboratories [21]. Compared to the normally growing *E. coli* ATCC 25922, *E. coli* 105X exhibits a slow growth phenotype (Fig 1). Genomic analysis revealed that this slow-growth phenotype arises from a complex adaptive mechanism involving nonsynonymous mutations in key structural genes, a significantly expanded repertoire of antibiotic resistance determinants, and a reprogrammed virulence strategy (Fig 3).

In this study, *E. coli* 105X was initially identified as *S. boydii* based on biochemical reactions, whereas MALDI-TOF MS unequivocally assigned it to *E. coli*, highlighting the risk of misidentifying invasive *E. coli* as *Shigella* by conventional phenotypic tests. Phylogenomic reconstruction and fastANI confirmed that *E. coli* 105X clusters with both *E. coli* and *Shigella* species, exhibiting ANI values ≥ 95% relative to *S. dysenteriae* SWHEFF 49, *S. sonnei* SE6−1, *S. boydii* ESBL-W3-2 and *S. flexneri* 2a str. 301 (ANI = 96.44%) (Fig 2C). *E. coli* and *Shigella spp*. share >99% 16S rRNA sequence identity and similar proteomic signatures, yet differ markedly in pathogenicity and clinical outcome [3,4]. Dif et al. reported ANI > 96% and dDDH > 70% between *E. coli*, supporting the re-classification of *Shigella* as an *E. coli* [5].

The slow-growth phenotype of *E. coli* 105X is not a simple growth defect but a multifaceted adaptation rooted in widespread genomic alterations. Enrichment analyses of nonsynonymous variants revealed pronounced perturbations in carbon and energy metabolism (Fig 3A). Metabolic bottlenecks, particularly within the purine nucleotide biosynthetic pathway, have been demonstrated to decelerate growth and simultaneously enhance β-lactam tolerance [9,22], suggesting that analogous lesions in *E. coli* 105X underlie its slow-growth and altered resistance phenotypes. Additionally, the over-representation of ABC transporters is noteworthy; these permeases govern iron and amino-acid uptake and are pivotal for UPEC fitness and virulence [23]. KEGG pathway enrichment identified mutations within CAMP-resistance and β-lactam-resistance modules, suggesting a potential role in evading host innate immunity and antibiotic challenge [22]. Alterations in amino acid metabolism are inferred to reprogram biosynthetic fluxes, promoting bacterial survival under nutrient deprivation [23,24]. Similarly, enrichment of fatty acid degradation and pyruvate metabolism genes indicates metabolic adaptation to oxidative and nutrient stress [25]. Finally, the accumulation of mutations in the flagellar assembly pathway suggests structural or regulatory modifications that could influence motility, biofilm formation and host colonisation, thereby modulating the bacterium’s environmental and host adaptability [24,25].

Crucially, we identified 29 specific genes directly implicated in growth regulation. Mutations in 11 flagellar assembly genes (e.g., *flgA*, *fliK*) likely disrupt structure assembly [26,27], diverting cellular energy (ATP) from proliferation to futile repair cycles [28,29]. Similarly, mutations in 12 fimbrial genes (e.g., *fimA*, *fimH*) and the peptidoglycan synthase gene *mrcB* are predicted to compromise biofilm integrity [30–33]. The *mrcB* mutation is particularly significant, as PBP1b deficiency is directly linked to prolonged doubling times [13], and impaired biofilms diminish nutrient sequestration, further attenuating growth [34]. Collectively, these lesions illustrate a sophisticated trade-off: *E. coli* 105X sacrifices rapid proliferation to reallocate energy towards stress survival, forming the foundation of its persistent phenotype.

Unlike the prototype UPEC strain CFT073, *E. coli* 105X jettisons enterobactin, P fimbriae, and α-haemolysin-factors that trigger robust immune responses [35,36]. Instead, it acquires *Yersinia*-type IV pili, lateral flagella, and an expanded ACE T6SS, potentially enhancing adherence, motility, and immune evasion [25,36–39]. A missense mutation in *fimH* may optimize this shift from biofilm formation to dissemination [32]. This re-engineered virulence suite is complemented by a formidable resistance portfolio. Over 80% of its 53 resistance genes encode efflux pumps (e.g., *acrA*, *acrB*, *tolC*), whose overexpression is synergistically regulated by global regulators (*marA*, *soxS*) [40,41] and potentiated by the slow-growth state, which reduces antibiotic target activity [9]^.^ Among these mechanisms, overexpression of the RND-type AcrAB-TolC efflux system not only facilitates the extrusion of β-lactams and fluoroquinolones but also reduces membrane permeability, thereby contributing to enhanced resistance to aminoglycosides [23]; *marR* and *acrR* products possess dual roles in target modification and efflux regulation [42,43]; Furthermore, the EvgA/EvgS and H-NS regulatory systems are known to induce distinct sets of eff pump genes [44]. The isolate’s broad resistance suggests that slow growth and high resistance are co-adaptive traits, enabling persistence under antibiotic and host pressures. The presence of ESBL genes CTX-M-5*5* and TEM-1 matches the predominance of CTX-M + TEM genotypes in clinical isolates [45]. This highlights the need for molecular-guided antimicrobial stewardship to limit the spread of multidrug-resistant strains.

Collectively, the slow-growing, multidrug-resistant clinical *E. coli* strain profiled in this study delivers robust evidence to optimize routine clinical pathogen identification pipelines. Our results further highlight the imperative to substitute conventional biochemical assays with more robust molecular platforms, including MALDI-TOF mass spectrometry and WGS.

## Conclusion

This case highlights that slow-growth *E. coli* strains can be misidentified as *S. boydii* by conventional biochemical profiling, underscoring the critical need for genomic or MALDI-TOF MS‑based identification in clinical microbiology. The delayed growth phenotype was attributed to mutations in genes involved in purine metabolism (*impA*, *purH*), cell wall integrity (*mrcB*), and motility (*flgA*, *fliK*, *fimH*), which collectively impair nucleotide synthesis, biofilm formation, and energy distribution. This adaptive trade-off may reflect a virulence-metabolism balance commonly observed in pathogenic Enterobacteriaceae. They suggest that energy-targeted therapeutic strategies, such as the combination of fosfomycin and efflux pump inhibitors, could be effective against multidrug-resistant pathogens exhibiting slow-growth phenotypes.

## Supporting information

S1 Fig Serological analysis of *E. coli* 105X. Shown are the serological test results of *E. coli* 105X (A) and the corresponding positive controls (B).
(TIF)

S2 Fig MALDI-TOF MS analysis of strain 105X. The MALDI-TOF MS results suggest that strain 105X is *E. coli*.
(TIF)

S1 Table Biochemical test results of *E. coli* 105X. The biochemical profile of *E. coli* 105X is shown alongside the expected results for *E. coli* and *S. boydii* as provided by the manufacturer’s reference database.
(DOCX)

S2 Table Quality-controlled data statistics of *E. coli* 105X. Listed are the total reads count, total base count, average read length, Q20 base count, Q20 base ratio, GC base batio, etc.
(DOCX)

S3 Table Growth-related gene mutations in *E. coli* 105X. Listed are the gene name, mutation type, impact level, annotation, and HGVS p.
(XLSX)
