# Supplementary material for: Isolation, identification, and slow-growth phenotype characterization of a clinically derived Escherichia coli from urinary tract infection
Source: PLoS One. 2026 Oct 29;21(10):e0358948. doi: 10.1371/journal.pone.0358948 (PMC13718956; doi:10.1371/journal.pone.0358948)
Supplement: Supplementary file 1 [file pone.0358948.s003.docx]

**S1 Table. Biochemical test results of *E. coli* 105X**

| **Biochemical** | ***E. coli* 105X Result** | ***E. coli***  **Result** | ***S. boydii***  **Result** |
| --- | --- | --- | --- |
| ARGININE-ARGININE-AMC | － | ＋ | － |
| GLUTARYL-GLYCINE-ARGININE-AMC | － | － | － |
| L-LEUCINE-AMC | ＋ | ＋ | － |
| L-PYROGLUTAMIC ACID-AMC | － | － | － |
| ACETATE | － | － | － |
| COLISTIN | － | － | － |
| MALONATE | － | － | － |
| 4MU-N-ACETYL-BD-GLUCOSAMINIDE | － | － | － |
| PNP-BD-GLUCOSIDE | － | － | － |
| BETA-GENTIOBIOSE | － | － | － |
| D-GALACTOSE | － | － | － |
| SORBITOL | － | － | － |
| L-ARABINOSE | － | ＋ | ＋ |
| MALTULOSE | － | － | － |
| ORNITHINE | － | ＋ | － |
| GLYCINE-PROLINE-AMC | － | － | － |
| L-ARGININE-AMC | － | － | － |
| L-PHENYLALANINE-AMC | － | － | － |
| L-TRYPTOPHAN-AMC | － | － | － |
| ADONITOL | － | － | － |
| D-MANNITOL | ＋ | ＋ | － |
| POLYMYXIN B | － | － | － |
| GAMMA-L-GLUTAMYL-NA | － | － | － |
| BIS (PNP) PHOSPHATE | － | － | － |
| DEXTROSE | ＋ | ＋ | ＋ |
| D-GLUCONIC ACID | ＋ | ＋ | － |
| SUCROSE | － | － | － |
| N-ACETYL-GALACTOSAMINE | － | ＋ | － |
| UREA | － | － | － |
| GLYCINE-AMC | － | － | － |
| L-GLUTAMIC ACID-AMC | ＋ | － | － |
| L-PROLINE-AMC | － | － | － |
| LYSINE-ALANINE-AMC | － | ＋ | － |
| CITRATE | － | － | － |
| ALPHA-KETOGLUTARIC ACID | － | ＋ | － |
| TIGLIC ACID | － | － | － |
| L-PROLINE-NA | ＋ | － | － |
| BETA-ALLOSE | － | － | － |
| D-FRUCTOSE | ＋ | ＋ | ＋ |
| D-MELIBIOSE | － | － | － |
| GALACTURONIC ACID | － | ＋ | － |
| METHYL-B-GLUCOSIDE | － | － | － |
| N-ACETYL-GLUCOSAMINE | ＋ | ＋ | － |
| ESCULIN | － | － | － |
