# Supplementary material for: Isolation, identification, and slow-growth phenotype characterization of a clinically derived Escherichia coli from urinary tract infection
Source: PLoS One. 2026 Oct 29;21(10):e0358948. doi: 10.1371/journal.pone.0358948 (PMC13718956; doi:10.1371/journal.pone.0358948)
Supplement: Supplementary file 2 [file pone.0358948.s004.docx]

**S2 Table. Quality-controlled data statistics of *E. coli* 105X**

| ***E. coli* 105X** | |
| --- | --- |
| Total Reads Count | 5936322 |
| Total Bases Count(bp) | 889403110 |
| Average Read Length(bp) | 149.82 |
| Q20 Bases Count(bp) | 870348652 |
| Q20 Bases Ratio(%) | 97.86% |
| Q30 Bases Count(bp) | 836827623 |
| Q30 Bases Ratio(%) | 94.09% |
| GC content(%) | 50.43% |
| Clean Reads(%) | 99.94% |
| Low Quality(%) | 0.00% |
| Too Many N(%) | 0.06% |
| Too short(%) | 0.00% |

Total Reads Count: The total number of all reads in the sample.

Total Base Count: The total number of all bases.

Average Read Length: The average sequence length.

Q20 Base Count: The number of bases with a quality above Q20.

Q20 Base Ratio: The ratio of bases with a quality above Q20.

GC Base Ratio: The GC content.

Clean Reads: Data after quality control.

Low Quality: In a read, 40% of the base quality is lower than Q15, or the paired-end reads have a length of less than 35 nt.

Too Many N: The proportion of bases that cannot be accurately identified in a sequence exceeds 1%.

Too short: Sequences with a remaining length below the minimum threshold (20 bp) after removing adapter sequences and trimming low-quality bases.
